# Documenting the Results of a Successful Partnership: A New Meningococcal Vaccine for Africa

**DOI:** 10.1093/cid/civ592

**Published:** 2015-11-09

**Authors:** Jean-Marie Okwo-Bele, F. Marc LaForce, Ray Borrow, Marie-Pierre Preziosi

**Affiliations:** 1 Department of Immunization, Vaccines and Biologicals, World Health Organization, Geneva, Switzerland; 2 Serum Institute of India, Ltd, Pune; 3 Vaccine Evaluation Unit, Public Health England, Manchester Royal Infirmary, United Kingdom; 4 Meningitis Vaccine Project, PATH, Ferney-Voltaire, France

**Keywords:** meningitis epidemics, meningitis vaccine project, partnership, meningococcal vaccine, vaccine development

The Meningitis Vaccine Project, a partnership between the World Health Organization (WHO) and PATH, an international nonprofit organization, existed from 21 June 2001 to 31 December 2014. The project had a single goal: the development, licensure, and introduction of a group A meningococcal conjugate vaccine in sub-Saharan Africa. It was successful and resulted in the WHO prequalification of 2 products, a PsA-TT 10-µg vaccine for use in mass vaccination campaigns among 1- to 29-year-olds, and a 5-µg vaccine for use in routine immunization programs among children <2 years of age. Since 2010, the 10-µg vaccine has been used to immunize 1- to 29-year-olds in large vaccination campaigns in countries of the sub-Saharan African meningitis belt. WHO has recommended introduction of the 5-µg vaccine into the routine childhood immunization program within 1–5 years following campaign completion.

Many lessons have been learned through the development, testing, and introduction of MenAfriVac (PsA-TT), and this collection of articles is an attempt at documenting results and lessons learned. Grouping articles on a single theme is an arbitrary exercise; the editors have chosen to group the purely serologic articles in a separate section. The initial section in general follows the historical procession from an idea that began in 1998 to the assessment of the impact of MenAfriVac introduction. A collection of 4 safety articles bridges sections 1 and 2. The editors have chosen to be inclusive so that readers may have a detailed view of the many steps required to develop and license a new vaccine. In addition to the group of articles that were included, there are previous publications, in particular, those describing the results of clinical trials using PsA-TT.

Many of development steps were used as important training opportunities. The article by Dellepiane et al is an excellent description of the regulatory challenges facing companies that wish to license a new vaccine [1]. The use of a fast track for prequalification and WHO's accelerated process for country registration greatly facilitated getting the vaccine properly registered.

Diomande's article on pharmacovigilance is particularly important given the all-too-common assertion that pharmacovigilance cannot be done in sub-Saharan Africa [2]. Similarly, the article by Wak et al clearly documents the safety of PsA-TT when given to pregnant women [3].

More than 217 million doses have been given from 2010 through December 2014, and group A *Neisseria meningitidis* has disappeared wherever the vaccine has been used. The vaccine's impact is well summarized in Diomande's article that amply documents vaccine impact, particularly in Chad and Burkina Faso [4].

Diallo et al describe the antibody persistence in 2- to 29-year-olds, demonstrating that antibodies at 1 and 4 years following immunization with PsA-TT persist significantly better than following polysaccharide vaccination. Four years following a single dose of PsA-TT, most individuals still had protective antibody titers [5]. Tapia et al demonstrated that in 12- to 23-month-old Africans, antibody levels remained significantly above baseline up to 5 years following vaccination, with the majority of children remaining protected [6].

An added benefit of immunizing with PsA-TT is the carrier protein tetanus toxoid (TT) itself. PsA-TT has been shown to generate robust tetanus serologic responses in 1- to 29-year-olds, similar to those expected after a booster dose of TT [7]. Of note, neonatal cases of tetanus have fallen by 25% in countries that completed PsA-TT campaigns in 1- to 29-year-olds.

No vaccine-related serious adverse events (SAEs) occurred during the 3 months of follow-up of 4004 healthy Malians vaccinated with a single dose of PsA-TT; in addition, rates of systemic reactions, adverse events, and SAEs were similar to those in the polysaccharide control vaccine group and were considered unrelated to vaccination [8].

This collection of articles offers a unique look into an important public health problem that has been controlled. Nonetheless, the need for continued enhanced meningitis surveillance in Africa remains a high priority because of the threat that 1 or more non-A meningococcal strains in Africa will assume epidemic proportions. PsA-TT's success has generated a new confidence that over time, and with the development and use of affordable polyvalent meningococcal conjugate vaccines, meningococcal disease may well be eliminated from sub-Saharan Africa.
